# Posterior Reversible Encephalopathy Syndrome in ALL

**DOI:** 10.15844/pedneurbriefs-29-7-7

**Published:** 2015-07

**Authors:** J. Gordon Millichap

**Affiliations:** 1 Division of Neurology, Ann & Robert H. Lurie Children's Hospital of Chicago, Chicago, IL; 2 Departments of Pediatrics and Neurology, Northwestern University Feinberg School of Medicine, Chicago, IL

**Keywords:** Encephalopathy, Leukemia, Hypertension, Seizures, Chemotherapy

## Abstract

Investigators from Soochow University, Suzhou, China, studied the possible pathogenetic mechanisms and treatment of posterior reversible encephalopathy syndrome (PRES) observed in 11 cases of pediatric acute lymphoblastic leukemia (ALL) after induction chemotherapy.

Investigators from Soochow University, Suzhou, China, studied the possible pathogenetic mechanisms and treatment of posterior reversible encephalopathy syndrome (PRES) observed in 11 cases of pediatric acute lymphoblastic leukemia (ALL) after induction chemotherapy. The clinical symptoms of PRES disappeared after appropriate treatment in most cases, even though induction chemotherapy continued. During the 1-year follow-up, no recurrence of PRES was observed. PRES should be recognized as an important complication of ALL that is reversible when diagnosed and treated early. Of the 11 children, 7 were boys and 4 girls. They were reviewed at an average age of 8.5 years (range, 5-14 years old). During the ALL inductive treatment (VDLD), 4 patients (36%) had an increase in blood pressure. Intrathecal chemotherapy included methotrexate, cytosine arabinoside, and dexamethasone. During the chemotherapy period (days 7 to 30) patients developed acute brain dysfunction, manifested by headache (10/11), epileptic seizure (7/11), visual impairment (6/11), disturbed consciousness (5/11), and ambulatory instability (2/11). Seizures were generalized convulsive, lasting 1 to 4 minutes. The EEG was abnormal in 9/11 cases, showing diffuse slow waves, usually in the occipital, temporal, and parietal lobe. The EEG repeated 4 weeks later was normal in all except one case. MRI within 2 days of onset revealed multiple lesions, mainly in the occipital, parietal, and frontal lobes. Chemotherapy was discontinued and treatment with mannitol, captopril, and valproic acid started. All patient lesions in the MRI shrunk after 2 weeks, and clinical symptoms of PRES disappeared completely within 2 to 4 weeks. Improvement in MRI lesions occurred later than clinical improvement. During 1-year follow-up, all patients continued treatment with ALL chemotherapy, and no PRES symptoms were observed. [1]

COMMENTARY. Drugs used in VDLD (vincristine, daunorubicin, L-asparaginase, dexamethasone) chemotherapy and intrathecal therapy are likely risk factors for development of PRES in children treated for ALL., the onset in the present series occurring 7 to 30 days after VDLD therapy [1]. A previous report by Kim et al [2] analyzed predisposing factors of 19 pediatric ALL patients who developed PRES; they had all received induction chemotherapy. Many factors have been considered as causative of PRES. Hypertension and toxic effects of various drugs used in therapy are most plausible. Since most chemotherapy is polytherapy, the involvement of a single drug is difficult to determine. Methotrexate neurotoxicity was implicated in a 15-year-old female child with ALL who presented with status epilepticus after receiving intrathecal methotrexate [3]. MRI showed reversible cortical and subcortical high intensity lesions consistent with the diagnosis of PRES. Early diagnosis and treatment of PRES in ALL patients is essential for a favorable prognosis. The most common presenting features of this syndrome are sudden arterial hypertension, pre-eclampsia, uremia, and immunosuppressive drug treatment [4]. Seizures are also of common occurrence.
